# The Effectiveness of Psychiatric Day Centres: Longitudinal Pilot Study

**DOI:** 10.1007/s10597-025-01541-1

**Published:** 2025-12-19

**Authors:** Sonja Mötteli, Léonie Strasser, Jenny Peracchi, Julia Häberli, Dirk Richter

**Affiliations:** 1 Centre for Psychiatric Rehabilitation, University Psychiatric Services Bern, Berne, Switzerland; 2 Psychiatric Hospital of the University of Zurich, Zurich, Switzerland; 3 Department of Psychology, University of Bern, Berne, Switzerland; 4 Department of Health Professions, Bern University of Applied Sciences, Berne, Switzerland; 5 University Hospital of Psychiatry and Psychotherapy, University of Bern, Berne, Switzerland

**Keywords:** Day centres, Daily routines, Severe mental illness, Psychiatric rehabilitation, Occupational rehabilitation

## Abstract

Day centres provide low-threshold support for people with severe and prolonged mental illness. However, their effectiveness has not yet been empirically evaluated. This study examines whether regular attendance at day centres improves mental health outcomes. Two surveys were conducted: a cross-sectional survey of 87 service users and a longitudinal study of 16 new admissions (assessments at baseline, one month, and three months after admission). Psychosocial participation limitations (IMET), quality of life (MANSA), symptom severity (SCL-K-9, PHQ-9), self-esteem (G-SISE) and self-efficacy (ASKU) were measured. Descriptive statistics and the Wilcoxon-signed-rank test were used for analysis. Three primary goals for attending a day centre were most important: daily routines, social contacts, and meaningful activities. Significant improvements (*p* < 0.05) were observed in all outcomes, with large effect sizes (r = > 0.5). This study provides initial evidence that the use of day centres supports recovery from mental illness by restoring key psychosocial functions.

## Introduction

People with mental disorders have a higher risk of long-term unemployment and social exclusion compared to the general population (Richter & Hoffmann, 2019). Unemployment causes additional mental distress through a loss of structure, activity, social contacts, economic resources, and other functions (Jahoda, 1981, 1982; Paul & Moser, 2009). Nevertheless, most people with mental disorders would like to have a paid job in the primary labour market (Adamus et al., 2024). However, they often face several internal barriers, such as a lack of confidence and illness-related cognitive and motivational problems, as well as external barriers, such as stigma (Blank et al., 2011).

For people with prolonged and severe mental illness who are not able to work and for whom even the requirements of sheltered workshops are too high, day centres are considered the lowest-threshold option for being meaningfully occupied (e.g., flexible working hours, no pressure). Day centres are non-medical, day-structuring services that offer long-term, low-threshold support for people with mental illnesses and focus mainly on aspects such as daily routines, meaningful occupations and leisure activities, social contacts and individual and practical support (Gruyters et al., 1997; Mötteli et al., 2024; Schene, 2004). Day centres do not have a theoretical foundation (Catty et al., 2005). However, their overarching aim of enhancing people’s mental health, social functioning, and personal recovery can be related to certain theories, such as the self-determination theory (Deci & Ryan, 2000), the recovery approach (Anthony, 1993) and the Jahoda theory about the value of work (Jahoda, 1981, 1982), to name a few. Concerning the latter, the day centres’ programmes address the latent factors ‘time structure’, ‘social contacts’, ‘collective purpose’, ‘status’, and ‘activity’, whose loss results from unemployment and affects mental health (Paul et al., 2023; Zechmann & Paul, 2019). Satisfying these latent needs through meaningful occupations and social contacts, day centres should contribute to recovery from mental illness and counteract the adverse effects of unemployment. However, although day centres are meaningful and helpful from the users’ point of view (Argentzell et al., 2012; Leufstadius, 2018; Mötteli et al., 2024), there are insufficient studies on their effectiveness (Catty et al., 2007). To our knowledge, no single longitudinal study has investigated the effects of day centres. Therefore, in this study, the goals and effects of the regular use of day centres on mental health parameters have been examined for quality and development purposes of a day centre service. Based on Jahoda’s theory (Jahoda, 1981, 1982) and a previous study, including focus groups with day centre users (Mötteli et al., 2024), the following parameters have been identified as key functions of day centres: improving social inclusion/participation, quality of life, severity of symptoms, self-esteem and self-efficacy. This pilot study examined whether these parameters are sensitive to changes (improvements) in a cohort of new admissions before, one month, and three months after day centre use.

## Materials and methods

### Setting

The day centres at the Centre for Psychiatric Rehabilitation of the University *“Psychiatric Services Bern (CPR UPD Bern)”* offer protected environments for people with severe mental health problems (mainly psychosis and affective disorders) who are receiving outpatient treatment and a disability pension and are unable to work in a sheltered workshop. The day centres of the *“CPR UPD Bern”* have been run at three different locations in *“Bern”* for over two decades and focus mainly on production-oriented activities (e.g., handcrafting articles), supplemented by weekly leisure afternoons. In June 2023, 160 individuals visited the day centre, of which one-third had a psychotic and another third had an affective disorder as a main psychiatric problem. Minimum attendance time is 2½ hours on at least one day per week, for which the users receive a recognition allowance. A lunch table is also offered. The day centres aim to enable social inclusion by providing daily routines, social interactions, and meaningful tasks.

## Study Design

This quality and development project included a complete sampling approach using a cross-sectional questionnaire addressing all service users (Survey 1) and an interview-based cohort study of the new admissions (Survey 2).

## Participants and Procedure

### Survey 1

During two weeks in June 2023, service users were asked to complete an anonymous online semi-quantitative questionnaire about their utilization, visiting reasons and satisfaction with the day centres. A trainee supervisor was instructed to help filling out the questionnaires (using smartphones or tables). The completion lasted about 20min, took place in the rooms of the day centres and was implemented using the software Unipark. After consenting, a total of *n* = 87 regular service users participated in the questionnaire (response rate of 54.4%); 25.0% were not available during the period of data collection (e.g., holiday, inpatient treatment); 14.4% could not participate due to poor mental health; 4.4% had insufficient German language skills; and 1.9% refused to participate.

### Survey 2

The survey was pretested with eleven users who had been visiting the day centre for between one and twelve months. From September 2023 to August 2024, *n* = 41 new admissions to the day centres were asked by their supervisors for study participation over three months about their psychosocial functioning and mental health. The service users’ contact details were given to the study team upon informed consent. The participants were interviewed personally by trainee psychologists (LS, JP and JH) in the first two weeks of their admissions (T0), after one month (T1) and after three months (T2). The interviews took place in a private space of the day centres and lasted about one hour. Data were collected using the software Unipark, and participants’ data were matched according to an anonymized identification code. In total, 16 participants (response rate of 39.0%) agreed to participate and took part in the first interview (T0); 24.4% could not be reached during the first two weeks of their admissions; 12.2% could not participate due to poor mental health; 4.9% had insufficient German language skills; and 19.5% refused to participate.

## Measures

The questionnaires were developed with the head of the day centres and a peer worker.

## Survey 1

### Sociodemographic Variables

Gender (male, female, diverse), age (years), education and housing (answer options as depicted in Table 1.) were assessed.

### Day Centre Utilization

Duration (‘How long have you been attending the day centre? Indicate years’), days of attendance per week, reasons or personal goals for visiting the day centre (open question), overall satisfaction with the day centre (‘0 = not satisfied at all’ to ‘10 = very satisfied’).

### Psychosocial Participation

Restrictions in participation in different dimensions of life were assessed using the validated IMET scale (index for assessing health impairments) ranging from ‘0 = no impairment’ to ‘10 = no activity possible anymore’ (Deck et al., 2011, 2015). The IMET is a self-assessment instrument for recording patient participation in nine areas of life: (1) activities of daily living (e.g., washing, eating), (2) activities at home (e.g., housework, gardening), (3) activities outside the home (e.g., shopping, driving around, doctor visits), (4) duties (e.g., cleaning up, care of others, work, school), (5) recreational activities (e.g., sports, hobbies, leisure time), (6) social activities (e.g., meeting friends, eating out, going to the theatre), (7) close relations (e.g., partner, family), (8) sexual life, (9) coping with stress and extraordinary strain (e.g., conflicts with family, stress at work). The ninth area (sexual life) was not included in this study due to many missing values in the pretests.

### Survey 2

The following variables were assessed at baseline (T0):

#### Sociodemographic Variables

Gender (male, female, diverse), age (years), education and housing (answer options as depicted in Table 1.) were assessed.

#### Day Centre Utilization

Days of attendance per week, reasons for visiting the day centre (open question), interest in getting a paid job in the primary labour market (yes, yes but only in future, no), and overall satisfaction with the day centre (0 = ‘not satisfied at all’ to 10 = ‘very satisfied’) was assessed at T1.

The following variables were assessed at baseline (T0), after one month (T1) and after three months (T2):

#### Psychosocial Participation and Functioning

Restrictions in participation in different dimensions of life were assessed using the validated IMET scale described in Survey 1. Quality of life was measured using the Manchester Short Assessment of Quality of Life (MANSA), which assesses satisfaction in various areas of life such as life as a whole, job, finances, friendships, leisure activities, accommodation, safety, physical and mental health (Priebe et al., 1999). It consisted of 12 subjective items, whereby the item on satisfaction with sex life was excluded for the same reasons as in the IMET. Responses were recorded on a 7-point Likert-type scale (0 = ‘completely dissatisfied’; 6 = ‘completely satisfied’).

#### Mental Health

Participants self-rated their mental health using two reliable, efficient, and valid scales. First, the nine-item Symptom Checklist (SCL-K-9) assessed psychopathological symptomatology over the past seven days on a five-point Likert-type scale. Higher scores indicate more severe symptoms (Prinz et al., 2013). Second, the nine-item Patient Health Questionnaire (PHQ-9) was used to assess the severity of depression in the past two weeks (Kroenke et al., 2001). Higher scores indicate more depressive symptoms (10–14 = mild depression, 15–19 = moderate depression, 20–27 = severe depression). In addition, the primary psychiatric diagnosis was subjectively assessed.

#### Self-esteem and Self-efficacy

Self-esteem was measured using the Single-Item Self-Esteem Scale (G-SISE) on a five-point Likert-type scale ranging from 0 = ‘strongly disagree’ to 4 = ‘strongly agree’ (Brailovskaia & Margraf, 2020). Self-efficacy expectations were measured using the reliable and validated Short Scale for Measuring General Self-Efficacy Beliefs (ASKU) consisting of three items on a five-point Likert-type scale ranging from 0 = ‘strongly disagree’ to 4 = ‘strongly agree’ (Beierlein et al., 2013).

### Analyses

The first author summarised the answers to the open question regarding the reasons for/goals of attending the institution into nine categories, which were then cross-checked by the second author (see Table 1.). Descriptive analyses were based on means (M) and standard deviations (SD) for continuous and frequencies for categorical variables. Scale scores, such as the mean of IMET, were calculated if at least 66% of the items were completed. Differences in socio-demographic variables between Sample 1 and Sample 2 were descriptively analyzed. Due to the small sample size, Wilcoxon signed-rank tests were calculated to examine differences (improvements) in variables of IMET, MANSA, SCL-K-9, PHQ-9, G-SISE, and ASKU among the 12 study completers of Sample 2 between T0 and T2. Related to the non-parametric results, the median (Mdn) was also reported. The significance level was set at 5% (one-sided). Effect sizes were calculated based on Rosenthal’s r (interpretation similar to Cohen’s r) using the formula r = z/√n. Analyses were conducted using the statistical programming language R (version 4.3.0; R Core Team, 2025).

## Results

### Participant Characteristics

Data from the 16 study participants (Survey 2) were compared to regular service users of the day centres (Survey 1). Study participants were more likely to be female and nearly 10 years younger compared to the regular day centre users. Overall, they reflect a fairly representative sample of newly admitted day centre users, considering the heterogeneity of the general day centre users (Table 1.). Service users of day centres represent a mixed population, including individuals differing in gender, age, education and housing situation and with different attendance goals. They perceive substantial restrictions in participation in different dimensions of life and visit their approved day centres one and a half days per week for one to four years. Three primary goals for visiting a day centre seem most important: establishing daily routines, experiencing social exchange and support and having a meaningful occupation.

**Table 1.** Characteristics of n = 87 regular service users (Survey1) and n = 16 study participants (Survey 2)

	**Survey 1**		**Survey 2**	
	**n or M**	**% or SD**	**n or M**	**% or SD**
Gender				
male	32	36.8	3	18.8
female	5	58.6	11	68.8
diverse	4	4.6	2	12.5
Age (years)	48.8	12.4	39.3	13.3
Education				
Not completed school education	1	1.2	0	0
Compulsory schooling	25	28.7	4	25.0
Vocational training	39	44.8	9	56.3
Matura (university entrance exam)	4	4.6	3	18.8
Higher vocational education	12	13.8	0	0
University	6	6.9	0	0
Housing situation				
Living alone (with or without support)	58	66.7	9	56.3
Living with family or friends	14	16.1	3	18.8
Residential care home / assisted living	11	12.6	4	25.0
Others	4	4.6	0	0
Years of attendance in the institution				
< 1 year	18	20.7	16	100
1-4 years	40	46.0	0	0
5-10 years	26	29.9	0	0
>10 years	3	3.4	0	0
Mean days of attendance per week	1.4	0.8	1.75	1.0
Reasons/goals for attendance in the institution				
Daily routines	28	32.2	10	62.5
Social exchange and support	26	29.9	9	56.3
Meaningful occupation	16	18.4	9	56.3
Stability and resilience	17	19.5	2	12.5
Participating in working life	11	12.6	3	18.8
Improving health	9	10.3	1	6.3
Preparing for the general labour market	9	10.3	3	18.8
No goals (attendance due to mental illness)	8	9.2	0	0.0
Improving self-confidence	6	6.9	1	6.3
General satisfaction with the day centre	8.1	1.5	7.9	1.1
unknown	1	1.1	1	6.3
IMET	4.3	2.3	4.2	2.0

Notes. Reasons/goals for attendance in the institution: multiple answers possible; questionnaires in Survey 1, personal interviews in Survey 2; percentages were rounded to the nearest 0.1 unit, resulting in +/-100%.

Of the 16 study participants, eight had an affective disorder, five had a psychotic disorder, and three had other/unknown disorders as the main psychiatric diagnosis. Four participants left the study prematurely for the following reasons: two hospital admissions, one subsequent solution and one extended holiday. The participants’ data at baseline, after one month and three months, are depicted as means and standard deviations in Table 2 as the data were almost normally distributed (*n* = 16). The values of IMET, MANSA, SCL-K-9, PHQ-9, G-SISE, and ASKU of the 12 study completers were compared with their values after three months and tested using the Wilcoxon signed-rank exact test. Significant improvements were found in all variables with strong effects: IMET (Mdn_T0_ = 4.50, Mdn_T2_ = 3.31, Z = −2.04, *p* = 0.020, *r* = 0.589), MANSA (Mdn_T0_ = 3.60, Mdn_T2_ = 3.91, Z = −1.84, *p* = 0.033, *r* = 0.531), SCL-K-9 (Mdn_T0_ = 1.17, Mdn_T2_ = 0.84, Z = −2.35, *p* = 0.008, *r* = 0.678), PHQ-9 (Mdn_T0_ = 1.00, Mdn_T2_ = 0.67, Z = −2.50, *p* = 0.005, *r* = 0.721), G-SISE (Mdn_T0_ = 1.00, Mdn_T2_ = 2.00, Z = −2.31, *p* = 0.018, *r* = 0.721), ASKU (Mdn_T0_ = 2.17, Mdn_T2_ = 2.50, Z = −2.11, *p* = 0.018, *r* = 0.609).

**Table 2:** Measurements at baseline, (T0), after one month (T1) and three months (T2)

	T0 (*n* = 16)		T1 (*n* = 15)		T2 (*n* = 12)	
	M	SD	M	SD	M	SD
IMET	4.2	2.0	4.2	1.8	3.6	1.9
MANSA	3.8	1.0	4.0	0.9	4.0	0.9
SCL-K-9	1.5	0.9	1.2	0.8	1.2	0.9
PHQ-9	1.2	0.6	1.0	0.5	0.9	0.7
G-SISE	1.4	1.2	1.7	0.9	2.1	0.9
ASKU	2.0	1.0	2.3	0.6	2.4	1.1

## Discussion

In this study, we examined for the first time whether day centre use is effective for improving users’ mental health. For this purpose, restrictions in participation (IMET), quality of life (MANSA), severity of symptoms (SCL-K-9, PHQ-9), self-esteem (G-SISE) and self-efficacy (ASKU) were examined in a cohort of newly day centre users before, one month and three months after they started in their day centres. The results showed significant improvements in all tested variables with large effect sizes (*r* > 0.5). This is remarkable, as stabilisation, in the sense of relapse prevention, is already a success for chronically mentally ill patients. In addition, a cross-sectional study was used to describe the population of day centre users and check for the study sample’s representativeness. Despite the small sample size, Sample 2 appears to be a representative group of day centre users.

Having a daily structure, social contacts and a meaningful occupation or activity were identified as the three most important goals of the participants and are in line with the latent functions of Jahoda’s theory (Jahoda, 1981, 1982). The day centre programme addresses the latent functions of work (e.g., ‘time structure’, ‘social contacts’, ‘collective purpose’, ‘status’, and ‘activity’), and it considers the psychosocial significance of work in terms of how it affects health (Paul et al., 2023). Previous studies on the subjective user perspective confirm that attending a day centre contributes to establishing a daily routine, increasing everyday activity and gaining social contacts (Argentzell et al., 2012; Leufstadius, 2018; Mötteli et al., 2024). In this way, the significant increase in self-esteem and self-efficacy is also consistent with the existing literature, which shows that engagement in day centres is similar to work and leads to an experience of competence (Argentzell et al., 2012; Leufstadius, 2018). In addition, Zechmann and Paul (2019) recommended including experience of competence as a sixth latent factor in Jahoda’s model as it positively affects mental health. Besides improving social inclusion and quality of life as explicit goals of day centres (Catty et al., 2005), improving mental health and work ability are also important functions of day centres (Mötteli et al., 2024). The results of this study indicate that the means of non-medical day centres, such as providing daily routines, meaningful occupations including leisure activities, social contacts and individual and practical support, can significantly help improve the user’s well-being.

Although the pilot study showed initial evidence for the effectiveness of day centres, several limitations exist. First, the data was only collected in one institution, meaning the results cannot be generalized to other settings. Second, the sample size was small because day centres are low-threshold services, and enrolling all new admissions in the study was challenging. In addition, one-fifth of the new admissions, probably those with higher impairment scores, refused to participate. Third, although we used established and validated scales, all mental health parameters were subjectively assessed and did not cover the full range of goals of the day centres. Third-party assessments should also be included in further studies.

Overall, the present findings suggest that non-medical day centre utilization can mitigate the negative consequences of losing the latent functions of work on psychological well-being and can contribute to recovery from mental illness. However, further longitudinal studies are needed to examine the effects over a longer period, as service users spend on average up to 4 years in day centres.

## Data Availability

Due to data sensitivity (belonging to a psychiatric institution), data cannot be shared publicly. If you are interested, please contact the corresponding author.
